# Clinical Trials for Artificial Intelligence in Cancer Diagnosis: A Cross-Sectional Study of Registered Trials in ClinicalTrials.gov

**DOI:** 10.3389/fonc.2020.01629

**Published:** 2020-09-15

**Authors:** Jingsi Dong, Yingcai Geng, Dan Lu, Bingjie Li, Long Tian, Dan Lin, Yonggang Zhang

**Affiliations:** ^1^Department of Lung Cancer Center, West China Hospital, Sichuan University, Chengdu, China; ^2^Department of Otorhinolaryngology, Head and Neck Surgery, West China Hospital, Sichuan University, Chengdu, China; ^3^Department of Periodical Press and National Clinical Research Center for Geriatrics, West China Hospital, Sichuan University, Chengdu, China; ^4^Chinese Evidence-Based Medicine Center, West China Hospital, Sichuan University, Chengdu, China

**Keywords:** clinical trial, diagnosis, artificial intelligence, ClinicalTrials.gov, cancer

## Abstract

**Objective:** Clinical trials are the most effective way to judge the merits of diagnosis and treatment strategies. The in-depth mining of clinical trial data enables us to grasp the application trend of artificial intelligence (AI) for cancer diagnosis. The aim of this study was to analyze the characteristics of registered trials on AI for cancer diagnosis.

**Methods:** Clinical trials on AI for cancer diagnosis registered on the ClinicalTrials.gov database were searched and downloaded. Statistical analysis was performed by using SPSS 20.0 software.

**Results:** A total of 97 registered trials were included. Of them, only 27 (27.8%) were interventional trials and 70 (72.1%) were observational trials. Fifteen (15.4%) trials had been completed. Fifty trials were in recruitment, and another 18 remained unrecruited. The number of cases included in the clinical trials tended to be large, 31 (32.0%) trials including samples ranging from 100 to 499 cases and 17 (17.5%) trials including samples ranging from 500 to 999 cases. Of the 27 interventional trials, only two trials reported trials' phase. Most (85.2%) interventional trials were for diagnosis, and a few (3.7%) were for the purpose of both the diagnosis and therapy of cancers. For the observational clinical trials, 46 (65.7%) were cohort studies, and 11 (15.7%) were case-only studies. Among the observational trials, 46 (65.7%) were prospective studies and 13 (18.6%) were retrospective studies. Among 97 trials, 37 (38.1%) involved colorectal cancer, 11 (11.3%) involved breast cancer, 43 (44.3%) were for imaging diagnosis, 33 (34.0%) were for endoscopic diagnosis, and 11 (11.3%) were for pathological diagnosis. For the interventional trials, 11 trials were parallel assignment (40.7%), and 14 were single group assignment (51.9%). Among the 27 interventional trials, 18 (66.7%) trials were performed without masking, 6 (22.2%) trials were performed with single masking, only 1 (3.7%) was performed with double masking, and 2 (7.4%) was performed with triple masking.

**Conclusion:** It appears that most registered trials on AI for cancer diagnosis are observational design, and more trials are needed in this field.

## Introduction

With the development of new computing methods combined with the availability of training data, the application of powerful mathematical algorithms in the field of artificial intelligence (AI) has been promoted. It is hard to define AI precisely. It has been suggested that a machine is intelligent if its working behavior is indistinguishable from that of a human being (1). In modern concepts, AI refers to the ability of machines to communicate, reason, and operate independently at work in a manner similar to that of humans. AI programs have been developed and applied in many medical areas, including diagnosis, treatment, drug development, and patient cares; in addition, there are increased researches regarding AI in various specialties, especially in cancer diagnosis (2–5).

Because cancer is still the leading cause of death worldwide (6), accurate diagnosis of cancers is essentially important. In terms of the imaging diagnosis of tumors (7) [i.e., pathological diagnosis (8) and endoscopic diagnosis (9)], the performance of AI is as good as that of human experts. In the era of big data, medical activities are accelerating to produce a vast amount of health-disease data (10). With the help of AI, doctors can provide medical services to patients more efficiently and accurately (11, 12). At present, the most critical problem for AI application in imaging is that there is no gold standard of AI for cancer diagnosis (12); thus, many researchers performed trials to assess AI for cancer diagnosis, because trials are the most effective way to judge the merits of diagnosis and treatment strategies (13). Most trials were registered in ClinicalTrials.gov, which is a public clinical trial registry platform jointly launched by the US Food and Drug Administration (FDA) and the US National Library of Medicine (NLM). Studying characteristics of registered trials will help to know the development of trials in specific filed. Up to now, there is no such study on AI for cancer diagnosis; thus, we performed the current study. The aim of this study was to analyze and summarize the characteristics of AI for cancer diagnosis. The in-depth mining of clinical trial data from ClinicalTrials.gov enables us to grasp the application trend of AI in cancer diagnosis earlier.

## Materials and Methods

### Inclusion Criteria and Exclusion Criteria

The inclusion criteria were registered trials on AI for cancer diagnosis; in each trial, cancer can be a single cancer or all kinds of cancer. The exclusion criteria were AI in purely therapeutic applications and incomplete registration information.

### Data Search

According to our previous studies (14, 15), we search the ClinicalTrials.gov on February 20, 2020, for trials on AI for cancer diagnosis. In case of missing any trials, we used the following words: artificial intelligence, deep learning, machine learning, etc. All searched results were downloaded. The data were updated on June 18, 2020.

### Trial Screening and Data Extraction

Two authors independently screened the trials according to the inclusion and exclusion criteria. In case of any disagreement, discussion was performed. And then, two authors independently extracted the data of the included trials. The following information was extracted: registered number, study type, start date, status of the trial, study result, study sample, participant age, primary sponsor, location, primary purpose, phases of the trial, allocation, intervention model, masking and intervention, and types of cancer.

### Data Analysis

The methodology of the study is similar to our previous study (14). This is a cross-sectional study, so a descriptive analysis was used to analyze the characteristics of registered trials. The outcomes included year, status of trials, study results, age, enrolment, sponsor, location, funding source, characteristics of study designs, type of cancer, and application method. Statistical analysis was performed by using SPSS 20.0 software. *P*-value <0.05 was considered statistically significant.

## Results

### The Characteristics of the Included Trials

On June 18, 2020, 884 results were searched from the ClinicalTrials.gov website. After a careful review of the clinical trial information, 787 results were excluded. Finally, a total of 97 trials were included in this study.

The characteristics of the included trials are shown in Table 1. Of the 97 trials, only 27 (27.8%) were interventional trials and 70 (72.1%) were observational trials. Fifteen (15.4%) trials were completed, the largest proportion of trials (50 trials) were in recruitment, and another 18 trials remained unrecruited. None of the trials had available results. Eighty-seven (89.7%) trials included subjects over the age of 18, and 10 trials (10.3%) included patients of all ages. The number of cases included in the trials tended to be large, with 31 (32.0%) trials including samples ranging from 100 to 499 cases and 17 (17.5%) trials including samples ranging from 500 to 999 cases. Universities were listed as the primary sponsor for 57 (58.8%) trials, hospitals for 30 (30.9%) trials, and industries for 10 (10.3%) trials. Of all the trials, 48 (49.5%) trials were performed in Asia, 15 in Europe, 33 in North America, and 1 in Australia.

**Table 1:** Characteristics of included trials.

**Characteristics**	**Number**	**Percentage (%)**
**Study type**		
Interventional	27	27.8
Observational	70	72.1
**Year**		
2007–2016	11	11.3
2017–2018	29	29.9
2019–2020	57	58.8
**Status**		
Completed	15	15.4
Recruiting	50	51.5
Active, not recruiting	6	6.1
Not yet recruiting	18	18.5
Unknown status	7	7.2
Withdrawn	1	1.0
**Study results**		
Has available results	0	0
No available results	97	100
**Participant age (y)**		
1 to older	10	10.3
Older than 18	87	89.7
**Enrollment**		
<99	17	17.5
100–499	31	32.0
500–999	17	17.5
>999	32	33.0
**Sponsor**		
University	57	58.8
Hospital	30	30.9
Industry	10	10.3
**Location**		
Europe	15	15.5
North America	33	34.0
Asia	48	49.5
Australia	1	1.0
**Funded by**		
Other		
Industry	8	8.2
Other and industry	6	6.2
Other	83	85.6

### Characteristics of the Study Design

The characteristics of interventional trials are displayed in Table 2. Of the 27 interventional trials, only two trials reported phase (1 in phase 1 and 1 in phase 3), and other trials did not report phases. Among all of the interventional trials, most (85.2%) of the interventional trials were for diagnosis, a few (3.7%) were for the purpose of both the diagnosis and therapy of cancers, 2 (7.4%) trials were for the primary purpose of screening, and 1 (3.7%) trial was for the primary purpose of device feasibility. Among all of the interventional trials, there were 10 randomized trials (37%) and 4 (14.8%) non-randomized trials, and 13 (48.1%) trials did not mention the allocation value. There were 11 parallel assignment (40.7%) and 14 single group assignment (51.9%). Among the 27 interventional trials, 18 (66.7%) trials were performed without masking, six (22.2%) trials were performed with single masking, only one (3.7%) was performed with double masking, and two (7.4%) was performed with triple masking.

**Table 2:** Study design elements of interventional trials.

**Characteristics**	**Number**	**Percentage (%)**
**Primary purpose**		
Diagnostic and treatment	1	3.7
Diagnostic	23	85.2
Screening	2	7.4
Device feasibility	1	3.7
**Phase**		
Phase 1	1	3.7
Phase 2	None	–
Phase 3	1	3.7
Phase 4	None	–
Not applicable	25	92.6
**Allocation**		
Randomized	10	37.0
Non-randomized	4	14.8
Missing value	13	48.1
**Intervention model**		
Parallel assignment	11	40.7
Sequential assignment	1	3.7
Crossover assignment	1	3.7
Single group assignment	14	51.9
**Masking**		
Single	6	22.2
Double	1	3.7
Triple	2	7.4
Without	18	66.7

Table 3 shows the characteristics of the observational trials (*n* = 70). For the observational trials, 46 (65.7%) were cohort studies, 11 (15.7%) were case-only studies, 1 (1.4%) was defined population, and 12 (17.1%) were other. Among the observational trials, 46 (65.7%) were prospective studies and 13 (18.6%) were retrospective studies.

**Table 3:** Study design elements of observational trials.

**Characteristics**	**Number**	**Percentage (%)**
**Observational model**		
Case-only	11	15.7
Cohort	46	65.7
Defined Population	1	1.4
Other	12	17.1
**Time perspective**		
Prospective	46	65.7
Retrospective	13	18.6
Cross- Sectional	3	4.3
Other	8	11.4

### Overview of Clinical Trials for Diagnosis

Table 4 shows the overview of trials for diagnosis. All 97 trials were designed for a variety of cancers, 37 trials (38.1%) were for colorectal cancer, and 11 (11.3%) were for breast cancer. Of the 97 trials, 43 (44.3%) were for imaging diagnosis, 33 (34%) for endoscopic diagnosis, and 11 (11.3%) for pathological diagnosis. To verify whether colonoscopy would be much more effective with the assistance of an automatic quality control system (AQCS), a prospective interventional trial was performed (NCT03622281). The enrolled patients were randomly assigned into the AQCS group and the control group who received colonoscopy without AQCS. An increased adenoma detection rate was seen in the AQCS group, which indicated that AQCS could practically improve the accuracy of colonoscopy (16).

**Table 4:** Overview of clinical trials in diagnosis.

**Characteristics**	**Number**	**Percentage (%)**
**Tumor types**		
Breast Cancer	11	11.3
Colorectal Cancer	37	38.1
Esophageal Cancer	3	3.1
Gastrointestinal Cancer	3	3.1
Glioma	6	6.2
Ovarian	2	2.1
Prostate	3	3.1
Pituitary	2	2.1
Lung Cancer	10	10.3
Skin Cancer	6	6.2
Other Cancer	14	14.4
**Application method**		
Endoscopy	33	34.0
Imaging	43	44.3
Pathology	11	11.3
Biomarker	6	6.2
Biopsy	4	4.1

Another trial with published results aimed to confirm whether a designed chatbot was not inferior to physicians regarding the satisfaction of breast cancer patients with the information provided (NCT03556813). Two groups of randomly assigned patients asked 12 predefined questions that were previously answered by a chatbot or a medical committee and received the response from either a chatbot or a physician. The chatbot group had higher success rates (69 vs. 64%) than the physician group, which showed noninferiority (*P* < 0.001) (17).

## Discussion

This study provided an assessment of the clinical trials registered on ClinicalTrials.gov about AI diagnosis in cancer. Most trials were observational, and only a few were interventional. Most trials were registered after 2017, indicating that the application of AI for cancer diagnosis was a new technology. More than half of the trials were in recruitment, and only one trial published available results. Most trials tended to be large sample size studies, and only a few studies had an expected sample size of <100. Most trials were sponsored by universities and hospitals. Notably, the vast majority (*n* = 48) of trials were conducted in Asia, with only 15 trials initiated in Europe and 33 trials initiated in North America. Most interventional trials used randomization, but most did not use blinding methods. Most observational trials were prospective design.

Ten trials were designed to diagnose lung cancer. In terms of the imaging diagnosis of lung cancer, it was reported that machine learning could predict the histological type of lung cancer through the imaging characteristics of PET/CT (18). In 2017, Song et al. (19) reported that CT imaging features could be used to predict the pathological type of micropapillary adenocarcinoma, but whether it could be recognized by AI had not been reported (20). In 2016–2017, Luo et al. (21) and Yu et al. (22) reported that the automatic analysis method of AI could perform pathological image analysis to predict the prognosis of patients with lung cancer. The CT diagnosis and pathological diagnosis of lung cancer are important prerequisites for the treatment of lung cancer (23–25). It was expected that AI could provide more functions for accurate diagnosis in the future.

In reviewing all included trials, the highest proportion trials were for colorectal cancer (37, 38.1%). This suggests that application of AI in diagnosis of colorectal cancer is a hot topic. Because the overall rate of missed polyps is as high as 22%, the associated colorectal cancer after colonoscopy is of concern (26). Computer-aided diagnosis (CAD) offers a promising solution for reducing the rate of missed diagnoses with colonoscopy (27). AI technology must address a number of important issues before it can be incorporated into routine clinical practice. The key stages for the implementation of CAD technology in routine colonoscopy have been detailed elsewhere, particularly by Mori et al. (28), who described the following steps: product development and feasibility studies, clinical trials, regulatory approval, and insurance reimbursement (29).

Eleven (11.3%) trials were for the pathological diagnosis of cancer. In 2019, Chen et al. (30) reported that thanks to ARM technology, AI can be integrated into the microscopic workflow to improve the efficiency and consistency of the microscopic inspection of biological specimens. The technology would be used to diagnose cancers. Among all the studies of AI in the field of cancer pathological diagnosis, the implementation in breast cancer was earlier and more mature (8), and AI diagnosis had an excellent application in the diagnosis of primary breast cancer and metastatic breast cancer (31). In terms of cancer imaging diagnosis, AI could also improve the specificity of diagnosis by integrating patient information and image analysis (32). In this study, we found that the diagnostic modes of lung cancer were mainly imaging diagnosis and pathological diagnosis. The diagnostic mode of colorectal cancer was mainly endoscopic diagnosis. The diagnostic modes of breast cancer were mainly imaging diagnosis and pathological diagnosis. In addition, both published studies used the randomization method, suggesting that the comparison of AI and traditional methods in tumor diagnosis was more operable in trials.

With the aid of AI, the detection rate of polyps and adenomas under endoscopy will be greatly improved (33). This apparent advantage, however, remains to be demonstrated in multicenter studies. The deficiency of AI in cancer diagnosis is also obvious. Chatbots designed to aid diagnosis could communicate with breast cancer patients like doctors, showing the potential to help doctors. But chatbot's questions are too routine to fully help doctors' diagnoses and decisions. The main purpose of this study is to understand the current situation of the application of AI in the field of medicine, which has a good hint to the scholars in related fields. Therefore, our study did not focus on the specific results of each trial.

The study had several limitations. First, not all studies were registered on ClinicalTrials.gov, which limits the application of the results in the future. However, ClinicalTrials.gov contained more than 80% of the trials on the World Health Organization's International Clinical Trials Registry Platform (34). Therefore, even if our study cannot cover all trials, it still reflected the mainstream of such trials. Second, the study analyzed data from clinical trials in which AI was used to diagnose cancer, but due to the short time span of the clinical trials, most clinical trials have not published results, so the analysis of results was insufficient. Third, this study included the application of AI in the diagnosis of all cancer types, but as only 97 trials were included, specific cancer types were not targeted for detailed analysis.

In conclusion, the current study presents the characteristics of registered trials on AI for cancer diagnosis. It suggested that more trials are needed to provide more evidence.

## Data Availability Statement

All datasets presented in this study are included in the article.

## Author Contributions

YZ designed the study. JD and YG provided the source for the study and edited the manuscript. JD, LT, and DLi searched, extracted, assessed, and analyzed the data and drafted the manuscript. JD, BL, and DLu analyzed the data. All authors contributed to the article and approved the submitted version.

## Conflict of Interest

The authors declare that the research was conducted in the absence of any commercial or financial relationships that could be construed as a potential conflict of interest.
